# Risk of *Mycoplasma pneumoniae*-related hepatitis in MP pneumonia pediatric patients: a predictive model construction and assessment

**DOI:** 10.1186/s12887-021-02732-x

**Published:** 2021-06-21

**Authors:** Yuna Bi, Yan Ma, Jinhua Zhuo, Lili Zhang, Liyan Yin, Hongling Sheng, Jie Luan, Tao Li

**Affiliations:** 1 Department of Pediatrics, Shandong Provincial Maternal and Child Health Care Hospital, Cheeloo College of Medicine, Shandong University, 238#, Jing 10 East Road, Jinan, Shandong China; 2 Department of Pediatrics, Cheeloo College of Medicine, Shandong Provincial Third Hospital, Shandong University, 12#, Wuyingshan Middle Road, Jinan, 250014 Shandong China; 3 Department of Clinical Laboratory, Cheeloo College of Medicine, Shandong Provincial Third Hospital, Shandong University, 12#, Wuyingshan Middle Road, Jinan, 250014 Shandong China; 4 Department of Pediatrics, Affiliated Hospital of Yangzhou University, 45#, Taizhou Road, Yangzhou, Jiangsu China; 5 Department of Infectious Diseases, Shandong Provincial Hospital Affiliated to Shandong First Medical University, 324#, Jing 5 Road, 250021 Jinan, China

**Keywords:** *Mycoplasma pneumoniae*-related hepatitis, Predictive model, Pediatric patients, *Mycoplasma pneumoniae* pneumonia

## Abstract

**Background:**

A predictive model for risk of *Mycoplasma pneumoniae* (MP)-related hepatitis in MP pneumonia pediatric patients can improve treatment selection and therapeutic effect. However, currently, no predictive model is available.

**Methods:**

Three hundred seventy-four pneumonia pediatric patients with/without serologically-confirmed MP infection and ninety-three health controls were enrolled. Logistic regressions were performed to identify the determinant variables and develop predictive model. Predictive performance and optimal diagnostic threshold were evaluated using area under the receiver operating characteristic curve (AUROC). Stratification analysis by age and MP-IgM titer was used to optimize model’s clinical utility. An external validation set, including 84 MP pneumonia pediatric patients, was used to verify the predictive efficiency. After univariate analysis to screen significant variables, monocyte count (MO), erythrocyte distribution width (RDW) and platelet count (PLT) were identified as independent predictors in multivariate analysis.

**Results:**

We constructed MRP model: MO [^10^9^/L] × 4 + RDW [%] – PLT [^10^9^/L] × 0.01. MRP achieved an AUROC of 0.754 and the sensitivity and specificity at cut-off value 10.44 were 71.72 and 61.00 %, respectively in predicting MP-related hepatitis from MP pneumonia. These results were verified by the external validation set, whereas it merely achieved an AUROC of 0.540 in pneumonia without MP infection. The AUROC of MRP was 0.812 and 0.787 in infants and toddlers (0–36 months) and low MP-IgM titer subgroup (1:160–1:320), respectively. It can achieve an AUROC of 0.804 in infants and toddler with low MP-IgM titer subgroup.

**Conclusions:**

MRP is an effective predictive model for risk of MP-related hepatitis in MP pneumonia pediatric patients, especially infants and toddlers with low MP-IgM titer.

**Supplementary Information:**

The online version contains supplementary material available at 10.1186/s12887-021-02732-x.

## Background

*Mycoplasma pneumoniae* (MP) has been considered as the predominant pathogenic bacteria species in community-acquired respiratory tract infection, which was detected in 30 % of pediatric community-acquired pneumonia and in over 50 % among children aged 5 years or older [1]. Recently, it was reported that extrapulmonary manifestations of MP pneumonia, such as aseptic meningitis, myocarditis, hepatitis and so on, occurred in up to 25 % of individuals [2], among which hepatitis is frequently ignored or at least downplayed. Actually, accumulated reports suggest that this MP-related hepatitis is commonly found in children and can manifest as asymptomatic elevation of liver enzymes, depressed multiple coagulation factors or cholestasis [3–9]. Therefore, ignoring of MP-related hepatitis will tend to delay its diagnosis and result in the possibility of serious liver damage during MP pneumonia.

MP pneumonia is recognized as a systemic inflammation characterized as extending beyond the lung and causing other co-morbidities including hepatitis [10]. Peripheral complete blood cell count (CBC) with differential can simultaneously change with ongoing inflammation process [11]. In terms of hepatitis, red cell distribution width (RDW), one of the parameters in peripheral CBC with differential, is frequently served as biomarker to predict disease process and mortality in autoimmune hepatitis, hepatitis B virus-related cirrhosis and acute-on-chronic liver failure [12–15]. Some scores incorporating the peripheral CBC with differential, such as neutrophil-to-lymphocyte ratio (NLR), platelet-to-lymphocyte ratio (PLR), monocyte-to-lymphocyte ratio (MLR) and systemic immune-inflammation index (S II) were also used to estimate the severity and outcome of patients with hepatitis B, C and E [16–19]. Therefore, a novel predictive model derived from parameters of peripheral CBC with differential can also be expected to predict MP-related hepatitis. Additionally, in view of the limited facilities available for monitoring hepatic function in community clinics, this predictive model may assist the community clinicians to screen the high-risk of MP-related hepatitis and improve the treatment. However, currently, there is no available predictive model for clinical practice and the insight of peripheral CBC with differential in predicting is also worth exploring further.

In current study, we firstly investigated whether these common inflammation markers (such as NLR, PLR, MLR and S II) can effectively distinguish MP-related hepatitis from MP pneumonia pediatric patients, and then we constructed a new predictive model with capable of predicting MP-related hepatitis. The specificity and efficacy of the predictive model were also verified by validation set and controls. To our knowledge, it is the first report that a novel model, derived from systemic inflammation markers, was constructed and applied to predict the MP-related hepatitis.

## Methods

### Participants

The retrospective cohort study enrolled 675 participants. Among these, 562 participants were enrolled from Shandong Provincial Third Hospital, Cheeloo College of Medicine, Shandong University from 2010.08 to 2017.08. One hundred and thirteen participants were collected from Affiliated hospital of Yangzhou University from 2013.06 to 2018.01. Exclusion criteria were as follows: (1) co-existent severe complication (e.g. jaundice, convulsions and dehydration et al.); (2) overlapping pathogenic infection; (3) receiving antibiotic treatment before admission; (4) having history of hepatotropic and/or non-hepatotropic virus infection (Hepatitis A, B, C and E virus, Epstein-Barr virus and cytomegalovirus), hereditary metabolic liver diseases (Wilson disease and Hereditary hemochromatosis) and drug-associated hepatitis; (5) insufficient medical records. The diagnosis of MP-related hepatitis was established from three aspects: (1) serologically-confirmed MP infection diagnosis: a highly elevated serological MP-IgM titer ≥ 1:320 (SeroDia™ Mayo MAG II microparticle agglutination (MAG) assay kit, Fujirebio, Japan). In cases with a lower MP-IgM titer (1:160), a second sample was examined after an interval of 2–3 weeks to confirm a 4-fold titer increase; (2) diagnosis of hepatitis: patients, who had serum alanine transaminase (ALT) and/or asparatate aminotransferase (AST) levels greater than the upper normal limits (ALT: 40 U/L (female) and 50 U/L (male); AST: 35 U/L (female) and 40 U/L (male)) were regarded as having hepatitis; (3) excluding liver biochemistry abnormalities caused by hepatotropic and/or non-hepatotropic virus infection, hereditary metabolic liver disease or drug-associated hepatitis [20, 21]. Because the elevation of gamma-glutamyl transpeptidase (GGT) and/or total bilirubin (TBIL), total bile acid (TBA) indicates that some damages may occur in the biliary capillary, these patients were also excluded.

### Study design

**Fig. 1:**
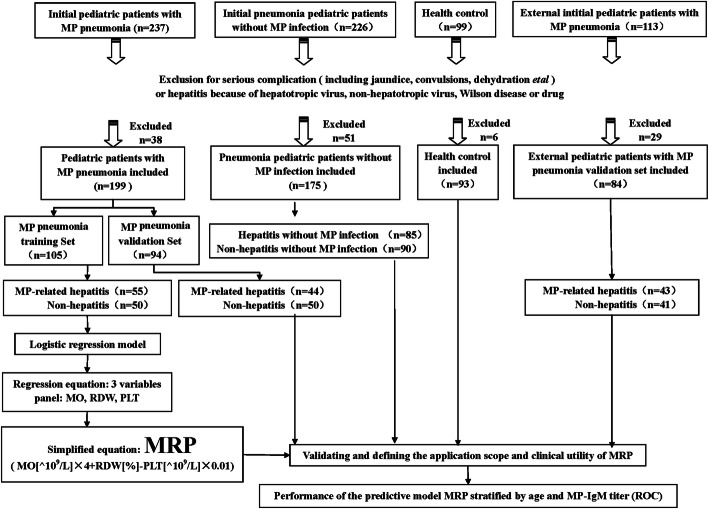
Participant enrollment and study design

As shown in Fig. 1 and 124 participants were excluded and 551participants were finally enrolled in the analysis, including 199 MP pneumonia pediatric patients, 175 pneumonia patients without MP infection, 93 healthy controls and 84 patients in an external validation set. Among 199 MP pneumonia patients, 99 patients were diagnosed as MP-related hepatitis and randomly divided into two sets: a training set (*n* = 55) and the validation set (*n* = 44), which was used to create and validate the model respectively. Otherwise, patient without presence of elevated serum ALT/ AST level were randomly classified as non-hepatitis set and served as control in both training (*n* = 50) and validating (*n* = 50) set at a ratio of 1:1 or so. One hundred and seventy-five pneumonia patients without MP infection, including 85 patients diagnosed as hepatitis and 90 patients with non-hepatitis, were used to define the specificity of the model. Ninety-three healthy control participants matching with patients by age and gender, accompanied with an external validation set (including 43 MP-related hepatitis and 41 non-hepatitis) were used to further validate the model’s application scope and predictive efficiency.

The following data were collected within 24 h at the time of admissions: demographic data, liver biochemistry, immunological marker and peripheral CBC with differential. The liver biochemistry test included ALT, AST, gamma-glutamyl transpeptidase (GGT), alkaline phosphatase (ALP), total bile acid (TBA), total protein (TP), albumin (ALB), globulin (GLO), total bilirubin (TBIL), direct bilirubin (DBIL), indirect bilirubin (IBIL) and total bile acid (TBA); immunological marker was MP-IgM titer; peripheral CBC with differential included WBC and their subtypes: lymphocytes, monocyte (MO), neutrophil, eosinophil, basophil proportion and count, RBC count and related information: hemoglobin, MCV, MCH, MCHC, RDW, PLT count and related information: platelet distribution width (PDW), mean platelet volume (MPV), platelet thrombocytocrit (PCT).

### Laboratory evaluation

All tests performance and results verification were conducted in Shandong Provincial Third Hospital, Cheeloo College of Medicine, Shandong University and Affiliated Hospital of Yangzhou University. Serological MP-IgM titer and peripheral CBC with differential were measured from the finger prick blood samples, while liver biochemistry test was measured in samples from the peripheral vein blood samples taken at the same time.

Serum MP-IgM titers were detected by SeroDia™ MAG assay (Fujirebio, Japan) [22]. Peripheral CBC with differential was determined using an automated MEK-7222 K hematology analyzer (Nihon Kohden, Japan). Liver biochemistry test including assessment ALT, AST, GGT, ALP, TBA, TP, ALB, GLO levels and TBIL DBIL IBIL were performed using the Beckman Coulter AU 5800 analyzer (Beckman Coulter Diagnostics, USA). Reference ranges for ALT were 7–40 U/L (female) and 9–50 U/L (male), for AST were 13–35 U/L (female) and 15–40 U/L (male), for GGT were 10–60 U/L, ALP were 45–125 U/L, TBA were 0–10 µmol/L, ALB were 40–55 g/L, GLO were 20–40 g/L, TBIL were 5.1–28 µmol/L, DBIL were 0–6.8 µmol/L and IBIL for 3.4–21.2 µmol/L.

### Statistical analysis

Statistical analysis was performed using SPSS software (version 22.0, SPSS Inc., USA). Data were expressed as Mean ± SD (or median, IQR) for quantitative variables and percentages for qualitative variables. The statistical significance between two groups was determined by *t* test or rank sum test for continuous variables and Chi-square test or Fisher’s exact test for categorical variables. The only endpoint in this study is the diagnosis of MP-related hepatitis. A two-sided *p* < 0.05 was considered statistical significance. Univariate analysis was firstly performed on all variables between patients with and without the study endpoint in training set. All selected variables were analyzed by multivariate analysis to assess the independent risk factors and were used to construct the predictive model. Predictive efficiency was evaluated using area under the receiver operating characteristic curve (AUROC) and verified in validation set and external validation set. Stratification analysis was used to seek the optimum application scope of the model. The optimal cut-off values was determined using the Youden index based on sensitivity and specificity [23].

## Results

### The participants’ characteristics

The characteristics of the participants are shown in Table 1 and Table S1. This study includes 238 male and 229 females with the median age being 43 months, ranging from 2 to 158 months. In the training set, the MP-related hepatitis group showed significantly lower PLT count (Mean ± SD : 290.13 ± 96.71 vs. 340.44 ± 89.79, *p* = 0.007). Other parameters, such MO count (median, interquartile range [IQR]: 0.6 (0.5, 0.8) vs. 0.4 (0.4, 0.6), *p* = 0.003), RDW (12.0 (11.4, 13.0) vs. 11.5 (11.1, 12.1), *p* = 0.004), PDW (17.34 ± 0.91 vs. 16.80 ± 1.00, *p* = 0.004) and MPV (5.35 ± 1.19 vs. 4.72 ± 0.99, *p* = 0.004) were significantly higher in the MP-related hepatitis group than the non-hepatitis group. No significant difference in MP-IgM titer, age, WBC count, lymphocyte count, neutrophil count, RBC count, hemoglobin and MCV et al. This followed the same trend as in the validation set and indicated that these two sets were well balanced in baseline characteristics.

**Table 1:** Characteristics of the pediatric patients with MP pneumonia (*n* = 199)

Variables (***n*** = 25 )	Training Set (***n*** = 105 )			Validation Set (***n*** = 94 )		
	MP-related Hepatitis ( ***n*** = 55 )	Non-Hepatitis (***n*** = 50 )	***P***	MP-related Hepatitis (n = 44 )	Non-Hepatitis (***n*** = 50 )	***P***
Gender						
Male, n (%)	28 (51.0)	31 (62.0)	0.253	20 (45.0)	16 (32.0)	0.181
Female, n (%)	27 (49.0)	19 (38.0)		24 (55.0)	34 (68.0)	
Anti-M.P IgM titer^a^						
Low titer, n (%)	35 (63.6)	26(52.0)	0.227	28 (63.6)	25 (50.0)	0.183
High titer, n (%)	20 (36.4)	24(48.0)		16 (36.4)	25 (50.0)	
Age, median (IQR) (Months)	50 (18,84)	50.5 (38.8,70.0)	0.717	64.5 (25.5,96.0)	60.5(41.0,80.0)	0.883
WBC, median (IQR) (^10^9^/L)	8.70 (6.4,10.7)	7.35 (6.48,9.13)	0.105	8.4 (6.7,11.3)	8.2 (6.18,9.38)	0.319
Neutrophil proportion (%)	53.46 ± 16.41	52.68 ± 16.78	0.810	52.26 ± 17.33	57.88 ± 13.06	0.083
Lymphocyte proportion (%)	38.00 ± 15.80	39.23 ± 16.31	0.695	38.69 ± 16.38	33.80 ± 13.03	0.111
MO proportion (IQR) (%)	6.90 (5.9,8.2)	5.90 (4.9.7.90)	0.139	6.5 (5.25,8.28)	6.7 (4.83,7.9)	0.471
Eosinophil proportion (IQR) (%)	0.50 (0.2,0.9)	0.60 (0.2,1.33)	0.203	0.75 (0.33,1.20)	0.7 (0.20,1.13)	0.276
Basophil proportion (IQR) (%)	0.30 (0.20, 0.90)	0.50 (0.30,1.00)	0.075	0.4 (0.2,0.6)	0.45 (0.3,0.7)	0.118
Neutrophil, median (IQR) (^10^9^/L)	4.40 (3.00,6.00)	4.00 (2.60,5.15)	0.175	4.4 (2.79,5.8)	4.25 (3.33,6.08)	0.625
Lymphocyte, median (IQR) (^10^9^/L)	2.80 (1.70,4.90)	2.95 (1.88,3.90)	0.790	3.19 (2.5,4.41)	2.8 (2.1,3.85)	0.150
MO, median (IQR) (10^9^/L)	0.6 (0.5,0.8)	0.4 (0.4,0.6)	0.003^*^	0.6 (0.4,0.87)	0.4 (0.3,0.6)	0.008^*^
Eosinophil, median (IQR) (^10^9^/L)	0.01 (0.00,0.10)	0.04 (0.00,0.10)	0.791	0.1 (0.00, 0.10)	0.06 (0.00,0.10)	0.273
Basophil, median (IQR) (^10^9^/L)	0.00 (0.00,0.04)	0.00 (0.00,0.10)	0.262	0.00 (0.00,0.04)	0.00 (0.00,0.10)	0.681
RBC count (^10^12^/L)	4.59 ± 0.41	4.52 ± 0.31	0.333	4.56 ± 0.42	4.59 ± 0.34	0.712
Hemoglobin (g/L)	127.40 ± 10.68	125.62 ± 9.50	0.371	125.05 ± 12.92	128.40 ± 9.18	0.147
Hematokrit (%)	37.88 ± 3.15	37.28 ± 2.63	0.295	37.39 ± 2.91	37.90 ± 2.54	0.365
MCV (fL)	82.90 ± 5.03	82.55 ± 3.79	0.695	82.65 ± 4.43	82.19 ± 6.15	0.684
MCH, median (IQR) (pg)	27.2 (26.35,28.70)	27.8 (27.05,29.05)	0.133	27.5 (26.48,28.28)	27.9 (27.1,28.9)	0.171
MCHC, median (IQR) (g/L)	331 (324,344)	335 (331,344)	0.071	336 (329.25,341)	338.5 (336,343)	0.054
RDW, median (IQR) (%)	12.0 (11.4,13.0)	11.5 (11.1,12.1)	0.002^*^	11.9 (11.4,12.58)	11.4(10.9,11.93)	0.001^*^
PLT (^10^9^/L)	290.13 ± 96.71	340.44 ± 89.79	0.007^*^	297.95 ± 99.43	337.48 ± 86.91	0.043^*^
PDW (fL)	17.34 ± 0.91	16.80 ± 1.00	0.004^*^	17.23 ± 0.80	16.83 ± 0.80	0.017^*^
MPV (fL)	5.35 ± 1.19	4.72 ± 0.99	0.004^*^	5.09 ± 0.94	4.70 ± 0.82	0.035^*^
PCT	0.16 ± 0.09	0.16 ± 0.04	0.878	0.16 ± 0.05	0.15 ± 0.04	0.245

NOTE. Continuous variables are expressed in the terms of Means ± SD for normal data or median and interquartile range for non-normal data. Comparison tests are performed using *t* test or rank sum test as appropriate. Categorical variables are expressed as n (%) and compared by Chi-square or Fisher exact tests

^a^Low titer was defined as 1:160-1:320 and the high titer as 1:640-1:1280

^*^p < 0.05 for significance

### Candidate predictors selected and predictive model training

**Fig. 2:**
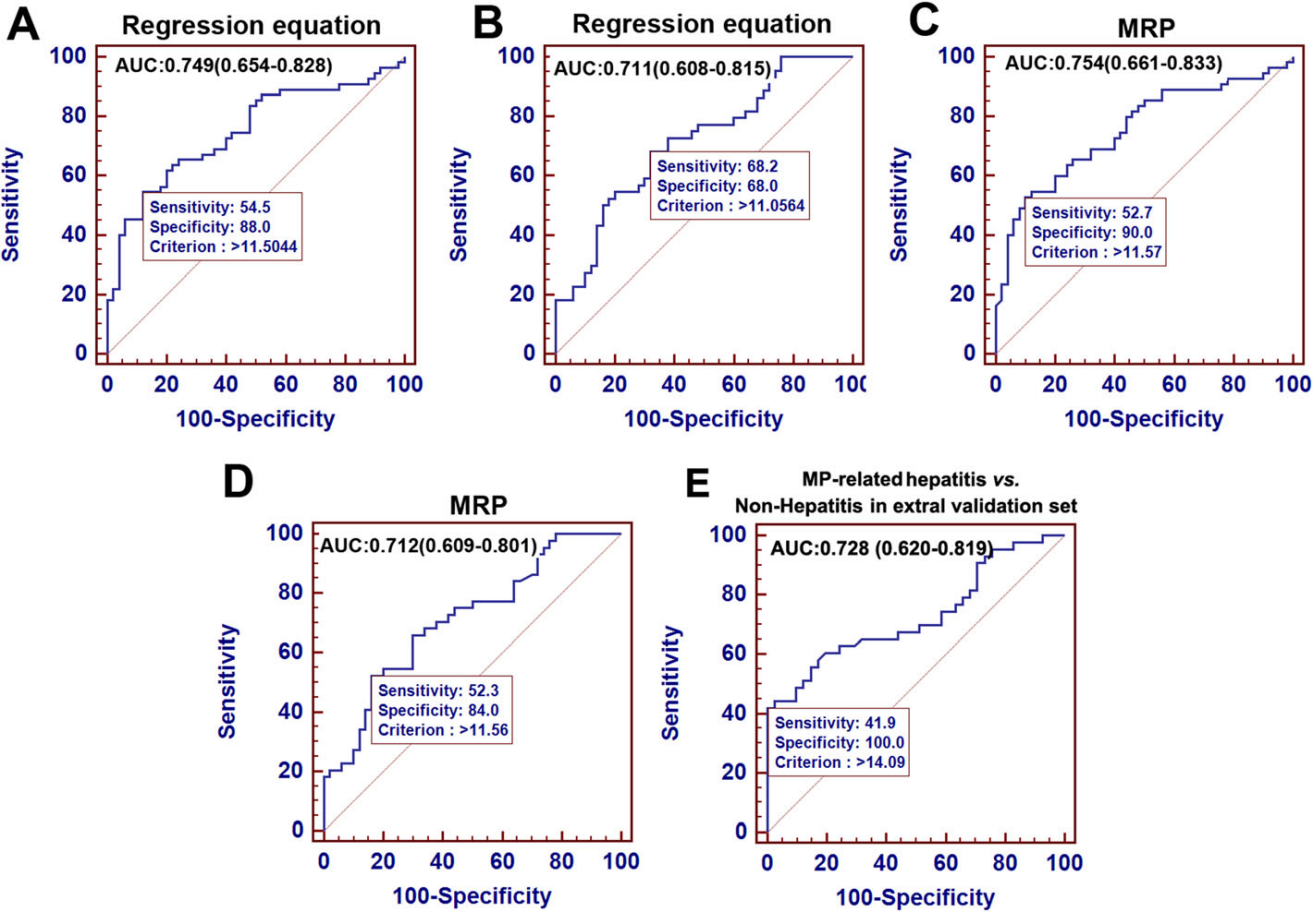
Receiver operating characteristic curve analysis for MP-related hepatitis diagnosis in training set, validation set and external validation set. Area under the curve (AUC) of Regression equation predicting for MP-related hepatitis in training set (**A**) and validation set (**B**); AUC of MRP predicting for MP-related hepatitis in training set (**C**) validation set (**D**) and in external validation set

The purposeful selection procedure began by a univariate analysis of each variable in the training set. A total of 7 variables (MP-IgM titer, MO count, WBC count, RDW, PLT count, PDW and MPV) were selected as determined by statistically significant from the original set of 25 variables in the training set using univariate analysis. Age was also selected by clinical experience. These 8 candidate predictors were further assessed using the multivariate logistic regression analysis. The MO count (odds ratio [OR]: 9.901, 95 % confidence interval [CI]: 1.730–56.650, *p* = 0.01), RDW (OR: 1.651, 95 % CI: 0.999–2.728, *p* = 0.05) and PLT count (OR: 0.995, 95 %CI: 0.990–1.000, *p* = 0.044) were significantly contributing to the prediction accuracy of the model (Table 2). Finally, the predictive model, consisting of 3-predictors with exact coefficient (Regression equation), was as follow: Regression equation = -5.532 + 2.293 × MO [^10^9^/L] + 0.501 × RDW [%] − 0.005 × PLT [^10^9^/L]. The receiver operating characteristic curves (ROC) were plotted in Fig. 2A and B with AUROC of 0.749 and 0.711, respectively. We simplified Regression equation into a new applicable equation and named as MRP: MO [^10^9^/L] × 4 + RDW [%] – PLT [^10^9^/L] × 0.01. AUROC of MRP in the training and validation set were 0.754 and 0.712, respectively (Fig. 2C and D). Both Regression equation and MRP were practically of similar accuracy, and MRP can be proposed as a novel predictive model for risk of MP-related hepatitis. In addition, AUROC of MRP in the independent external validation set was 0.728, which also confirms its predictive power (Fig. 2E).

**Table 2:** Univariate and multivariate analysis of variables in Training set of the MP pneumonia pediatric patients

Variables	MP-related Hepatitis ***vs*** Non-Hepatitis in Training set					
	Univariate	Multivariate^**a**^				
	***P***	B	S.E.	Odds ratio	95% CI	***P***
Intercept	-	-5.532	3.313	-	-	0.095
Age, median (IQR) (Months)	0.300	-	-	-	-	-
Anti-M.P IgM titer	0.028^*^	-	-	-	-	-
MO, median (IQR) (^10^9^/L)	0.008^*^	2.293	0.890	9.901	1.730-56.650	0.010
WBC, median (IQR) (^10^9^/L)	0.084^**§**^	-	-	-	-	-
Neutrophil l, median (IQR) (^10^9^/L)	0.545	-	-	-	-	-
Lymphocyte, median (IQR) (^10^9^/L)	0.193					
RDW, median (IQR) (%)	0.008^*^	0.501	0.256	1.651	0.999-2.728	0.050
PLT count (^10^9^/L)	0.010^*^	-0.005	0.003	0.995	0.990-1.000	0.044
PDW (fL)	0.006^*^	-	-	-	-	-
MPV (fL)	0.006^*^	-	-	-	-	-

NOTE. ^a^Logist(P)=-5.532+2.293×Mon[×10^9^/L]+0.501×RDW[%]-0.005×PLT [×10^9^/L]

^*^p < 0.05 for significance; ^**§**^*p* = 0.084 for marginal significance

### Specificity and clinical utility of MRP

**Fig. 3:**
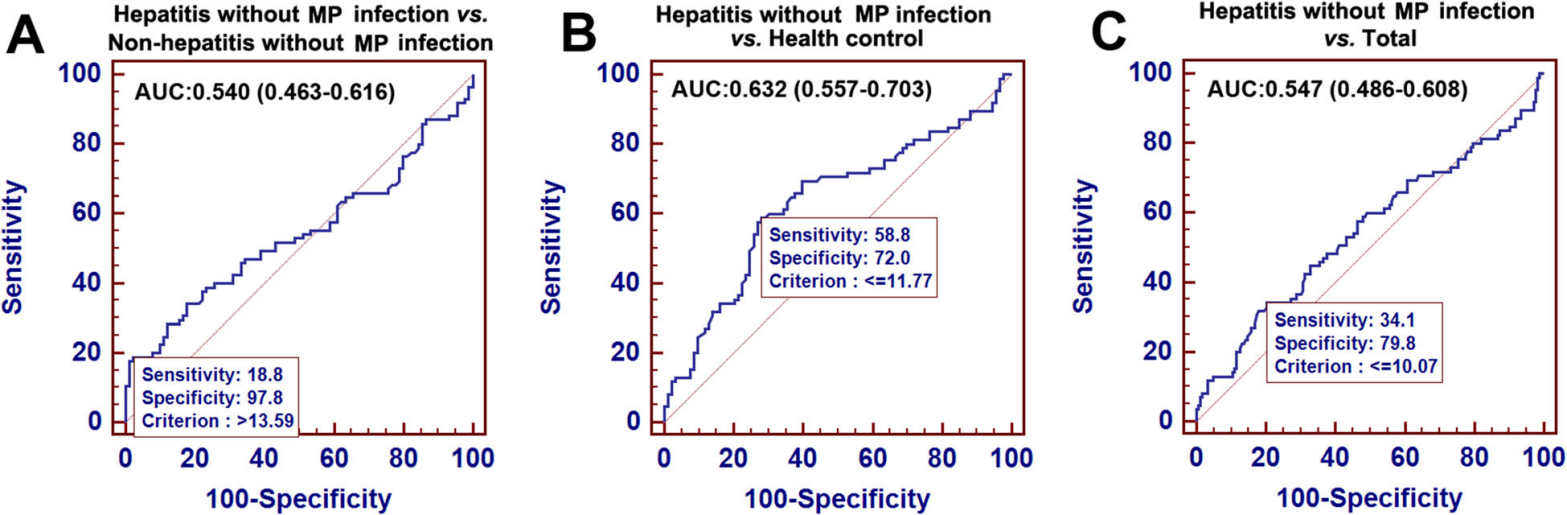
Receiver operating characteristic curve analysis of MRP for hepatitis in participants without MP infection and health control. Area under the curve (AUC) of MRP predicting hepatitis in participants without MP infection (**A**), health control (**B**) and the total of them (**C**)

Due to the derivation of the new predictive model MRP, the specificity needs to be defined carefully to guide MRP clinical application in pediatric pneumonia patients. Pneumonia pediatric patients without MP infection and health controls were introduced to test the specificity of MRP. As shown in Fig. 3A, MRP was inefficient for distinguishing the hepatitis from pneumonia patients without MP infection with an AUROC of only 0.540 (95 % CI: 0.463–0.616). Although, a slight improvement of MRP predictive efficacy, AUROC of 0.632 (95 % CI: 0.557–0.703) was obtained in distinguishing hepatitis from health controls (Fig. 3B), MRP predictive efficacy in hepatitis returned to be inefficient (with an AUROC of 0.547, 95 %CI: 0.486–0.608) from the combining non-hepatitis without MP infection and health controls (Fig. 3C). These results indicated that MRP applied only to predict hepatitis in MP pneumonia pediatric patients rather than pneumonia caused by other pathogens.

Based on the ROC curves of MRP, the optimized cut-off value for prediction was set 10.44 to screen MP-related hepatitis (Table 3). Using cut-off value of 10.44 in both training and validation set, 71.72 % of MP pneumonia patients with MRP > 10.44 were verified as MP-related hepatitis (i.e., positive predictive value (PPV) = 64.55 %), whereas an MRP ≤ 10.44 has a specificity 61.00 % and a negative predictive value (NPV) of 68.54 %. In the training set, for patients with MRP > 10.44, 40 of 60 (66.67 %) participants were MP-related hepatitis. Simultaneously, for patients with MRP ≤ 10.44, 30 of 45 (66.67 %) participants were non-hepatitis, only 15 (27.27 %) MP-related hepatitis was falsely classified. Meanwhile, the cut-off value for MAP was verified in the validation set. Similarly, in the higher MRP group (> 10.44), 31 of 50 (62.00 %) participants were MP-related hepatitis. while in the lower MRP group (≤ 10.44), 31 of 44 (70.45 %) participants were non-hepatitis, only 13 (29.55 %) MP-related hepatitis was classified incorrectly.

**Table 3:** Accuracy of MRP in predicting MP-related Hepatitis and Hepatitis without MP infection in different sets of the pediatric patients

	MRP cut-off value	Hepatitis	Non-hepatitis	Sensitivity	Specificity	PPV	NPV
**Total**^a^ **(*N*** **= 199)**	> 10.44	71	39	71.72%	61.00%	64.55%	68.54%
	≤10.44	28	61				
**Training Set (*N*** **= 105)**	> 10.44	40	20	72.73%	60.00%	66.67%	66.67%
	≤10.44	15	30				
**Validation Set (*N*** **= 94)**	> 10.44	31	19	70.45%	62.00%	62.00%	70.45%
	≤10.44	13	31				
**Pneumonia pediatric patients without MP infection (*N*** **= 175)**	> 10.44	55	59	64.70%	34.44%	48.25%	50.82%
	≤10.44	30	31				

^a^Consisting of the training set and validation set

### Optimum application scope of MRP

**Fig. 4:**
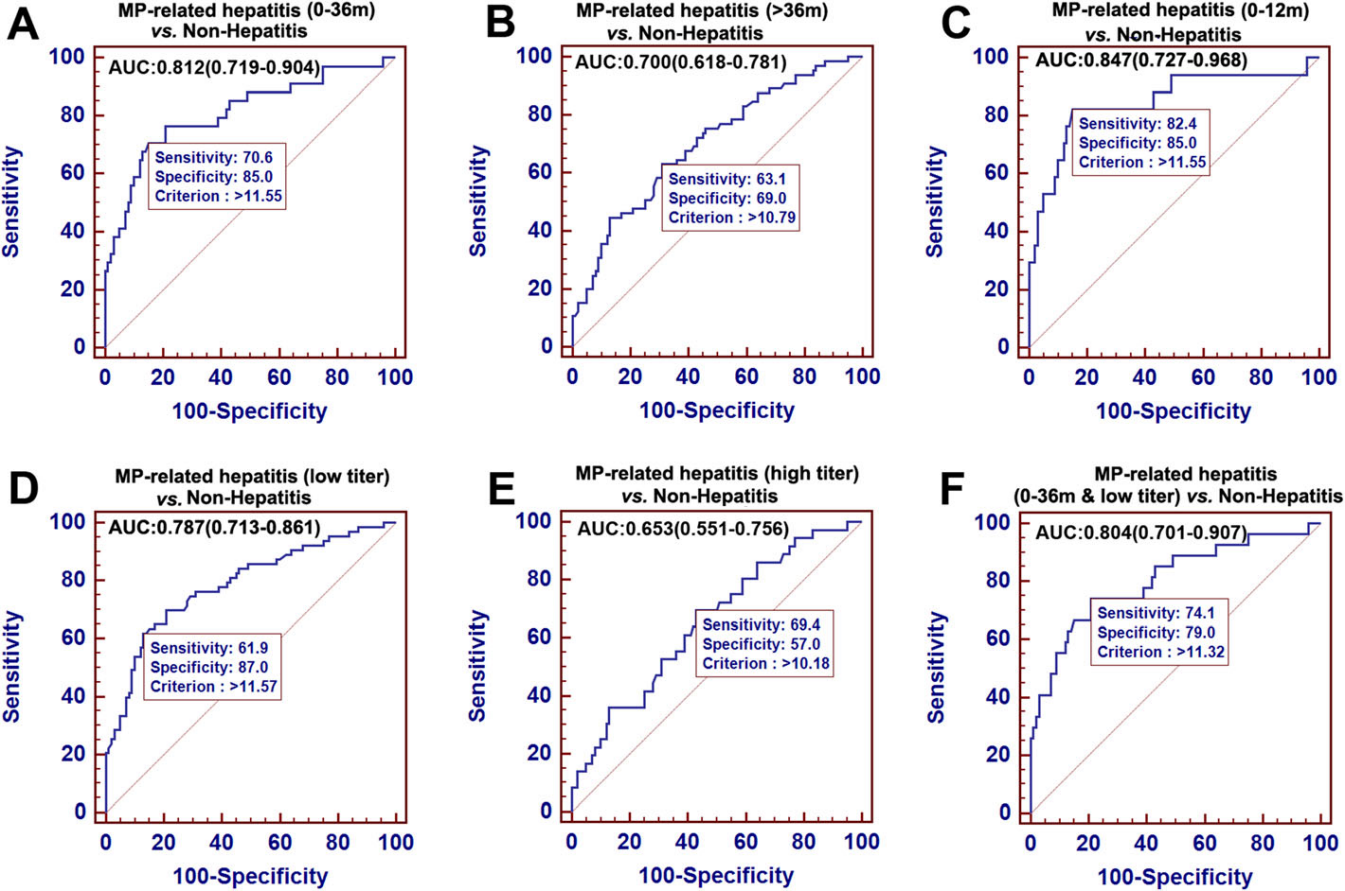
Receiver operating characteristic curve analysis of MRP for MP associated hepatitis diagnosis in different age and MP-IgM titer subgroups. Area under the curve (AUC) of MRP predicting MP-related hepatitis in whole set of participants with MP infection in infants and toddlers (age, 0-36 months) (**A**), preschooler and school-age child (age, > 36 months) (**B**) and infants (age, 0-12 months) (**C**); AUC of MRP predicting MP-related hepatitis in the low MP-IgM titer subgroup (1:160-1:320) (**D**), high MP-IgM titer subgroup (1: 640-1:1280) (**E**) and in infants and toddlers (age, 0-36 months) with low MP-IgM titer (1:160-1:320) (**F**)

To optimize MRP clinical application, stratification analysis by age and MP-IgM titer was performed to improve its diagnosis efficiency in MP-related hepatitis. As detailed in Table S2, stratification analysis by age and MP-IgM titer was performed to improve the predictive efficacy of MRP. Participants were commonly stratified in four subgroups: infants (0–12 months), toddlers (0–36 months), preschoolers (36–60 months) and school-ager (> 60 months). MRP predictive efficacy was strongest in the 0–12 (AUROC = 0.847, 95 % CI: 0.727–0.968) and 0–36 (AUROC = 0.812, 95 % CI: 0.719–0.904) months old subgroups (Fig. 4A, B and C). Meanwhile, the patients were also divided into low (1:160–1:320) and high (1:640–1:1280) MP-IgM titer subgroup. AUROC of MRP in low titer subgroup (0.787, 95 % CI: 0.713–0.861) was considerably greater than in high MP-IgM titer subgroup (0.653, 95 % CI: 0.551–0.756) (Fig. 4D and E). Moreover, MRP can achieve an AUROC of 0.804 (95 % CI: 0.701–0.907) in infant and toddler (0–36 months) with low MP-IgM titer (1:160–1:320) subgroup, which indicates the optimum application scope for MRP (Fig. 4F).

## Discussion

MP-related hepatitis, one of the extrapulmonary manifestations of MP pneumonia, has been receiving more attention recently [7], but for the clinical practice purposes, too little attention is paid for predicting risk of the MP-related hepatitis, especially in MP pneumonia pediatric patients. In this study, these common inflammation markers (including NLR, PLR, MLR and S II) were shown as unable to predict MP-related hepatitis from MP pneumonia patients (data not shown). We constructed a novel predictive model, MRP, had comparable accuracy for predicting MP-related hepatitis in MP pneumonia pediatric patients (AUC = 0.754), especially in infants and toddlers with low MP-IgM titer (AUC = 0.804). The performance of MRP was also validated in an external validation set with similar accuracy (AUC = 0.728).

Currently, it is recognized that MP-related hepatitis is closely relative to the systemic inflammatory response induced by MP infection [10]. Therefore, a comprehensive model composed of parameters derived from peripheral CBC with differential has potential to predict MP-related hepatitis. Among the MRP model, RDW has been identified as a biomarker in monitoring disease course and predicting the severity of hepatitis, such as autoimmune hepatitis [12], hepatitis B-related hepatitis [13, 14, 24–26] and nonalcoholic steatohepatitis [27]. RDW levels are usually elevated with progressive liver inflammation. A possible mechanism is that increased inflammatory cytokines in peripheral circulation and liver, such as TNF-α, IL-1β and IL-6, suppress erythrocyte maturation and accelerate the entry of newer, larger reticulocytes into the peripheral circulation, therefore causing the increased RDW [28, 29]. Platelet and MO are also included in the MRP model. It was reported that MLR was correlated with risk of death in patients with severe exacerbation of chronic hepatitis B [18] and can be used in the diagnosis of bacterial infection patients [11]. RDW-to-platelet ratio was a useful indicator for hepatic fibrosis regardless of etiology and can reflect the severity of hepatitis [30]. Therefore, these three parameters are all common biomarkers involved with systemic inflammation and hepatitis. Their combination in the MRP would offer lots of potential advantages in the prediction of MP-related hepatitis, which is caused by MP pneumonia inducing systemic inflammation.

In this study, we enrolled pneumonia patients without MP infection (including hepatitis and non-hepatitis) and healthy control to evaluate the specificity of the MRP model. The low AUC value (only 0.540, 95 % CI: 0.463–0.616) suggested that MRP can not apply to screen the hepatitis in these pneumonia patients without MP infection, which in turn ensured that MRP merely can be used to screen hepatitis in MP pneumonia patients. The specificity of MRP application shows some advantages in MP-related hepatitis diagnosis and is more suitable for clinical using. Moreover, we provided an optimal application scope of MRP: the infants and toddlers patients (0–36 months) with low MP-IgM titer (1:160–1:320). Although, we found the largest AUC (0.847, 95 % CI: 0.727–0.968) in the infants group (0–12 months), the relatively small-size of positive infants participants (*n* = 17) impacted the credibility of the MRP diagnosis efficiency. When we extended to include toddlers patients (age, 0–36 months), the positive participants (*n *= 34) size could sufficiently suppose the credibility of AUC (0.812, 95 % CI: 0.719–0.904).

Although, some MP-related hepatitis patients (25.00 % in training set and 29.54 % in validation set) were eventually misclassified by the MRP model, this simple model was also an useful method to screen the high-risk of MP-related hepatitis in MP pneumonia pediatric patients, with positive predictive value of 64.55 % and negative predictive value of 68.54 % at 10.44 cut-off point. Actually, as a novel simple noninvasive predictive model, MRP has a fairly high diagnostic efficiency, which can achieve an AUC of 0.804 in the optimum application scope. The misdiagnosed patients can receive liver biochemistry test to exclude eventually and the price can be affordable. However, enhancing the sensitivity of MRP is still needed.

Macrolide are widely used as the first-line antibacterial agents in MP pneumonia treatment [31]. However, macrolide-induced hepatotoxicity, which showed a significantly elevated liver enzyme values or even cholestatic hepatitis, had been reported [32, 33]. Pre-existing MP-related hepatitis may aggravate liver injury. Hence, it is necessary to evaluate the high-risk of MP-related hepatitis in MP pneumonia pediatric patients before the antibiotics application. The score of MRP, which can be calculated using only three parameters (MO, RDW and PLT) from the peripheral CBC with differential, can be used as “warning-signal” and provide a reference for selecting antibiotic and rational drug use through timely prediction of MP-related hepatitis. However, in our opinion, hepatitis would not be a contraindication to the use of macrolide antibiotics, the first-line antibacterial for MP infection, during the treatment of MP pneumonia. On the contrary, effective control of MP infection by macrolide antibiotics is critical for the recovery of MP-related hepatitis. After all, the possible hepatotoxicity induced by macrolide is not common in the real world [34]. With monitoring of the change of liver biochemistry and timely hepatoprotective treatment, it is reasonable that the possible hepatotoxicity induced by macrolide antibiotics can be reduced or even avoided.

**Fig. 5:**
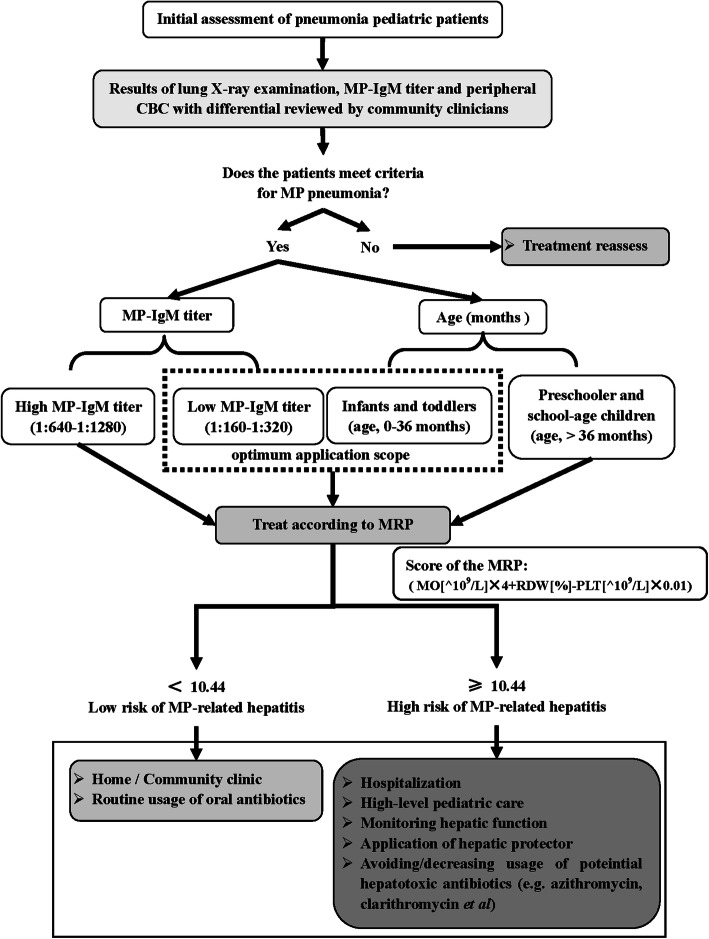
Flow chart of prediction and management of MP-related hepatitis in MP pneumonia pediatric patients in community clinics

With the facility of finger prick blood sampling, rapid measurement and reliable results, peripheral CBC with differential and MP-IgM titer measurement are commonly available in community clinics. Here the following flow chart can be suggested (see detail in Fig. 5): (i) community clinicians diagnose the MP pneumonia by means of lung X-ray examination and serum MP-IgM titer measurement. Meanwhile, the peripheral CBC with differential was also measured; (ii) the score of the MRP was calculated; (iii) if the score of MRP is greater than 10.44, especially in the low MP-IgM titer of infants and toddlers, it is highly recommended for high-level pediatric care (e.g. monitoring hepatic function, avoiding/decreasing usage of potentially hepatotoxicity antibiotics and so on). Otherwise, they can receive routine usage of oral antibiotics and care at home. Considering the limited facilities in community clinics and the difficulty in collecting peripheral vein blood samples in pediatric patients, it is convenient for community clinicians to evaluate the risk of MP-related hepatitis and help to select treatment and improve the therapeutic effect.

Our study has several limitations. Although a total of 551 participants from two medical centers were eventually enrolled for development and validation of MRP model, our model also needs to be further validated as yet with large prospective studies from multicenter. Additionally, this model developed from Asian race pediatric patients. Our result may not be representative of other race patients in general. We encourage our model to undergo validation in other races and also in centers outside China.

## Conclusions

The MRP model was identified as an effective predictive model for risk of MP-related hepatitis in MP pneumonia pediatric patients, especially in infants and toddlers (age, 0–36 months) with low MP-IgM titer (1:160–1:320). What’s more, we also provide a flow chart of prediction and management of MP-related hepatitis in MP pneumonia pediatric patients for more convenience in clinical application.

## Supplementary Information

**Additional file 1: Table S1. **Characterististics of the pneumonia pediatric patients without MP infection (*n* = 175 ), the external pediatric patients with MP pneumonia validation set (*n* = 84 ) and health control (*n* = 93 ).

**Additional file 2: Table S2. **ROC analysis of MRP for MP-related hepatitis diagnosis in different patients’ age and MP-IgM titer subgroups.

## Data Availability

The datasets used and/or analyzed during the current study are available from the corresponding author on reasonable request.

## Abbreviations

MP: *Mycoplasma pneumoniae*
MO: Monocyte
RDW: Red cell distribution width
PDW: Platelet distribution width
MPV: Mean platelet volume
PCT: Platelet thrombocytocrit
WBC: White blood cell
RBC: Red blood cell
IQR: Interquartile range
CI: Confidence interval
PPV: Positive predictive value
NPV: Negative predictive value
HAV: Hepatitis A virus
HBV: Hepatitis B virus
HCV: Hepatitis C virus
HEV: hepatitis E virus
EB: Epsten-Barr virus
CMV: Cytomegalovirus
ROC: Receiver operating characteristic
AUROC: Area under the receiver operating characteristic curve
NLR: Neutrophil-to-lymphocyte ratio
PLR: Platelet-to-lymphocyte ratio
MLR: Monocyte-to-lymphocyte ratio
S II: Systemic immune-inflammation index
ALT: Alanine transaminase
AST: Asparatate aminotransferase
GGT: Gamma-glutamyl transpeptidase
ALP: Alkaline phosphatase
TP: Total protein
ALB: Albumin
GLO: Globulin
TBIL: Total bilirubin
DBIL: Direct bilirubin
IBIL: Indirect bilirubin
TBA: Total bile acid

## References

[1] Atkinson TP, et al. Epidemiology, clinical manifestations, pathogenesis and laboratory detection of *Mycoplasma pneumoniae* infections. FEMS Microbiol Rev. 2008;32:956–73. 10.1111/j.1574-6976.2008.00129.x 18754792

[2] Saraya T (2017). *Mycoplasma pneumoniae* infection: Basics. J Gen Fam Med.

[3] Chen CJ (2004). *Mycoplasma pneumoniae* infection presenting as neutropenia, thrombocytopenia, and acute hepatitis in a child. J Microbiol Immunol Infect.

[4] Grullich C (2003). Acute *Mycoplasma pneumoniae* infection presenting as cholestatic hepatitis. J Clin Microbiol.

[5] Jujaray D (2018). Pattern and Significance of Asymptomatic Elevation of Liver Enzymes in Mycoplasma Pneumonia in Children. Clin Pediatr (Phila).

[6] Chang JH (2008). A case of acute hepatitis with *Mycoplasma pneumoniae* infection and transient depression of multiple coagulation factors. Yonsei Med J.

[7] Poddighe D (2020). *Mycoplasma pneumoniae*-related hepatitis in children. Microb Pathog.

[8] Song WJ (2017). Pediatric *Mycoplasma pneumoniae* Infection Presenting with Acute Cholestatic Hepatitis and Other Extrapulmonary Manifestations in the Absence of Pneumonia. Pediatr Gastroenterol Hepatol Nutr.

[9] Park SJ (2012). Fulminant and Fatal Multiple Organ Failure in a 12-Year-Old Boy With *Mycoplasma pneumoniae* Infection. Allergy Asthma Immunol Res.

[10] Narita M (2010). Pathogenesis of extrapulmonary manifestations of *Mycoplasma pneumoniae* infection with special reference to pneumonia. J Infect Chemother.

[11] Naess A (2017). Role of neutrophil to lymphocyte and monocyte to lymphocyte ratios in the diagnosis of bacterial infection in patients with fever. Infection.

[12] Zeng T (2018). Noninvasive indices for monitoring disease course in Chinese patients with autoimmune hepatitis. Clin Chim Acta.

[13] Fan X (2018). Association of red blood cell distribution width with severity of hepatitis B virus-related liver diseases. Clin Chim Acta.

[14] Zhang M (2017). Value of Red Cell Distribution Width in Assessing the Severity of Hepatitis B Virus-Related Decompensated Cirrhosis. Clin Lab.

[15] Jin L (2017). Clinical Usefulness of Measuring Red Blood Cell Distribution Width in Patients with Hepatitis B Virus-Related Acute-On-Chronic Liver Failure. Clin Lab.

[16] Pokora Rodak A (2018). Neutrophil-lymphocyte ratio and mean platelet volume as predictive factors for liver fibrosis and steatosis in patients with chronic hepatitis B. Ann Agric Environ Med.

[17] Wu J, et al. RDW, NLR and RLR in predicting liver failure and prognosis in patients with hepatitis E virus infection. Clin Biochem. 2019;63:24–31. 10.1016/j.clinbiochem.2018.11.012 30502317

[18] Wu W (2018). Characteristics of systemic inflammation in hepatitis B-precipitated ACLF: Differentiate it from No-ACLF. Liver Int.

[19] He Q, et al. The Relationship between Inflammatory Marker Levels and Hepatitis C Virus Severity. Gastroenterol Res Pract. 2016;2016:2978479. 10.1155/2016/2978479 PMC5206414 28090206

[20] Daxboeck F (2005). Elevated serum alanine aminotransferase (ALT) levels in patients with serologically verified *Mycoplasma pneumoniae* pneumonia. Clin Microbiol Infect.

[21] Shin SR (2012). Clinical characteristics of patients with *Mycoplasma pneumoniae*-related acute hepatitis. Digestion.

[22] Liu FC (2008). Do serological tests provide adequate rapid diagnosis of *Mycoplasma pneumoniae* infection?. Jpn J Infect Dis.

[23] Youden WJ (1950). Index for rating diagnostic tests. Cancer.

[24] Xu WS (2015). Red blood cell distribution width levels correlate with liver fibrosis and inflammation: a noninvasive serum marker panel to predict the severity of fibrosis and inflammation in patients with hepatitis B. Medicine.

[25] Wang H (2016). Red blood cell distribution width and globulin, noninvasive indicators of fibrosis and inflammation in chronic hepatitis patients. Eur J Gastroenterol Hepatol.

[26] Lan F, et al. Increased Red Cell Distribution Width is Strong Inflammatory Marker of Liver Diseases in a Guangxi Population. Clin Lab. 2017;63:389–98. 10.7754/Clin.Lab.2016.160626 28182351

[27] Zhou WJ (2019). Association between red cell distribution width-to-platelet ratio and hepatic fibrosis in nonalcoholic fatty liver disease: A cross-sectional study. Medicine.

[28] Lippi G, et al. Relation between red blood cell distribution width and inflammatory biomarkers in a large cohort of unselected outpatients. *Arch Pathol* Lab Med. 2009;133:628–32. 10.5858/133.4.628 19391664

[29] Krintus M (2017). Critical appraisal of inflammatory markers in cardiovascular risk stratification. Crit Rev Clin Lab Sci.

[30] Yuyun D (2019). Predictive value of the red blood cell distribution width-to-platelet ratio for hepatic fibrosis. Scand J Gastroenterol.

[31] Cao B (2017). Overview of antimicrobial options for *Mycoplasma pneumoniae* pneumonia: focus on macrolide resistance. Clin Respir J.

[32] Martinez MA (2015). Clinical and histologic features of azithromycin-induced liver injury. Clin Gastroenterol Hepatol.

[33] Ellison CA, et al. Acute Hepatocellular Injury Associated With Azithromycin. J Pharm Pract. 2021;34:1–4. 10.1177/0897190019894428 31928122

[34] Principi N (1999). Comparative Tolerability of Erythromycin and Newer Macrolide Antibacterials in Paediatric Patients. Drug Saf.

