# Supplementary material for: Risk of Mycoplasma pneumoniae-related hepatitis in MP pneumonia pediatric patients: a predictive model construction and assessment
Source: BMC Pediatr. 2021 Jun 21;21:287. doi: 10.1186/s12887-021-02732-x (PMC8218438; doi:10.1186/s12887-021-02732-x)
Supplement: Supplementary file 1 — Additional file 1: Table S1. Characterististics of the pneumonia pediatric patients without MP infection (n = 175 ), the external pediatric patients with MP pneumonia validation set (n = 84 ) and health control (n = 93 ). [file 12887_2021_2732_MOESM1_ESM.docx]

**Table S1** Characterististics of the pneumonia pediatric patients without MP infection ( n = 175 ), the external pediatric patients with MP pneumonia validation set ( n = 84 ) and health control ( n = 93 )

| **Variables ( n = 25 )** | **Pneumonia pediatric patients without MP infection**  **( n = 175 )** | **External pediatric patients with MP pneumonia validation set ( n = 84 )** | **Health control**  **( n = 93 )** |
| --- | --- | --- | --- |
| Gender |  |  |  |
| Male, n (%) | 34 (40.0) | 49(58.3) | 56 (60.0) |
| Female, n (%) | 51 (60.0) | 35 (41.7) | 37 (40.0) |
| Age, median (IQR) (Months) | 13 (6, 45.5) | 15.5 (6.75, 36) | 46 (22.5, 75) |
| WBC, median (IQR) (^10^9^/L) | 8.70 (6.75, 10.45) | - | 7.5 (6.40, 9.35) |
| Neutrophil proportion (%) | 42.80 ± 19.24 | - | 43.13 ± 14.27 |
| Lymphocyte proportion (%) | 48.78 ± 18.38 | - | 47.14 ± 15.36 |
| MO proportion (IQR) (%) | 6.80 (5.10, 9.10) | - | 6.50 (5.30, 8.20) |
| Eosinophil proportion (IQR) (%) | 0.70 (0.25, 1.30) | - | 2.0 (0.95, 3.15) |
| Basophil proportion (IQR) (%) | 0.40 (0.22, 0.68) | - | 0.50 (0.30, 0.70) |
| Neutrophil, median (IQR) (^10^9^/L) | 3.30 (2.20, 5.00) | - | 3.25 (2.25, 4.08) |
| Lymphocyte, median (IQR) (^10^9^/L) | 3.90 (2.53, 5.60) | - | 3.30 (2.48, 4.55) |
| MO, median (IQR) (10^9^/L) | 0.50 (0.40, 0.80) | 0.66 (0.49,0.94) | 0.52 (0.38, 0.64) |
| Eosinophil, median (IQR) (^10^9^/L) | 0.10 (0.00, 0.10) | - | 0.14 (0.06, 0.27) |
| Basophil, median (IQR) (^10^9^/L) | 0.00 (0.00, 0.09) | - | 0.04 (0.02, 0.05) |
| RBC count (^10^12^/L) | 4.56 ± 0.49 | - | 4.59 ± 0.48 |
| Hemoglobin (g/L) | 119.58 ± 12.98 | - | 125.54 ± 14.80 |
| Hematokrit (%) | 36.15 ± 3.64 | - | 36.67 ± 4.14 |
| MCV (fL) | 79.60 ± 6.21 | - | 80.09 ± 6.23 |
| MCH, median (IQR) (pg) | 26.55 (25.13, 27.55) | - | 27.90 (26.65, 29.00) |
| MCHC, median (IQR) (g/L) | 329.00 (321, 335) | - | 343 (337, 348.5) |
| RDW, median (IQR) (%) | 11.90 (11.25, 12.30) | 12.9 (12.40, 13.80) | 13.5(11.60, 14.05) |
| PLT count (^10^9^/L) | 312.05 ± 93.44 | 355.21 ± 131.76 | 293.34 ± 74.92 |
| PDW (fL) | 17.37 ± 1.13 | - | 17.96 ± 0.82 |
| MPV (fL) | 5.34 ± 1.03 | - | 6.08 ± 0.64 |
| PCT | 0.17±0.06 | - | 0.18 ± 0.05 |
