# Supplementary material for: Risk of Mycoplasma pneumoniae-related hepatitis in MP pneumonia pediatric patients: a predictive model construction and assessment
Source: BMC Pediatr. 2021 Jun 21;21:287. doi: 10.1186/s12887-021-02732-x (PMC8218438; doi:10.1186/s12887-021-02732-x)
Supplement: Supplementary file 2 — Additional file 2: Table S2. ROC analysis of MRP for MP-related hepatitis diagnosis in different patients’ age and MP-IgM titer subgroups. [file 12887_2021_2732_MOESM2_ESM.docx]

**Table S2** ROC analysis of MRP for MP-related hepatitis diagnosis in different patients’ age and MP-IgM titer subgroups

| **Stratification by Age and MP-IgM titer** | **AUC (95%CI)** | | |
| --- | --- | --- | --- |
|  | **Training set** | **Validation set** | **Total^a^** |
| MP pneumonia (Age)-1^b^ |  |  |  |
| MP associated hepatitis (0 - 12 m) *vs.* non-hepatitis | 0.816(0.626-1.000) | 0.889(0.756-1.000) | 0.847(0.727-0.968) |
| MP associated hepatitis (13 - 60 m) *vs.* non-hepatitis | 0.793(0.675-0.911) | 0.683(0.533-0.833) | 0.756(0.665-0.847) |
| MP associated hepatitis (> 60 m) *vs.* non-hepatitis | 0.690(0.553-0.828) | 0.676(0.542-0.809) | 0.684(0.589-0.779) |
| MP pneumonia (Age)-2^c^ |  |  |  |
| MP associated hepatitis (0 - 36 m) *vs.* non-hepatitis | 0.834(0.714-0.955) | 0.766(0.613-0.919) | 0.812(0.719-0.904) |
| MP associated hepatitis (> 36 m) *vs.* non-hepatitis | 0.705(0.589-0.821) | 0.689(0.571-0.807) | 0.700(0.618-0.781) |
| MP pneumonia (MP-IgM titer)^d^ |  |  |  |
| MP associated hepatitis (low titer ) *vs.* non-hepatitis | 0.823(0.724-0.922) | 0.730(0.612-0.848) | 0.787(0.713-0.861) |
| MP associated hepatitis (high titer) *vs.* non-hepatitis | 0.634(0.489-0.779) | 0.680(0.535-0.825) | 0.653(0.551-0.756) |
| MP pneumonia (Age & titer) ^e^ |  |  |  |
| MP associated hepatitis (0 – 36 m & low titer) *vs.* Non-hepatitis | 0.804(0.701-0.907) | | |

**NOTE**

**^a^** Consisting of the training set and validation set;

**^b^** The participants were stratified as infants (age, 0-12 months), as toddlers and preschoolers (age, 13-60 months) and as school-age child (age, > 60 months);

**^c^** as infants and toddlers (age, 0-36 months) and as preschooler and school-age child (age, > 36 months);

**^d^** Low titer was defined as 1:160 -1:320 and the high titer as 1:640 ~1:1280;

**^e^** The pediatric patients were simultaneously fitted with age (age, 0- 36 months) and low titer (1:160 - 1:320).
